# Mixed adenoneuroendocrine carcinoma with loss of HER2 positivity after trastuzumab-based chemotherapy for HER2-positive gastric cancer: a case report

**DOI:** 10.1186/s40792-020-0774-7

**Published:** 2020-01-08

**Authors:** Hiromi Nagata, Hironori Tsujimoto, Yoshihisa Yaguchi, Keita Kouzu, Yujiro Itazaki, Yusuke Ishibashi, Satoshi Tsuchiya, Takao Sugihara, Nozomi Ito, Manabu Harada, Shinsuke Nomura, Yoshitaka Utsumi, Hideyuki Shimazaki, Yoji Kishi, Hideki Ueno

**Affiliations:** 1 Department of Surgery, National Defense Medical College, 3-2 Namiki, Tokorozawa, 359-8513 Japan; 2 Department of Pathology, National Defense Medical College, 3-2 Namiki, Tokorozawa, 359-8513 Japan

**Keywords:** Trastuzumab-based chemotherapy, Gastric cancer, Conversion surgery, Liver metastasis, Prognosis

## Abstract

**Background:**

Trastuzumab (T-mab)-based chemotherapy is a standard regimen for human epithelial growth factor 2 (HER2)-positive gastric cancer. However, some patients have demonstrated a change in HER2 status after T-mab-based treatment of breast cancer. We report a rare case of mixed adenoneuroendocrine carcinoma with loss of HER2 positivity after T-mab-based chemotherapy for HER2-positive gastric cancer.

**Case presentation:**

A 60-year-old man presented with a mass of the upper abdomen, which was diagnosed as adenocarcinoma with a HER2 score of 3+ by endoscopic biopsy. He received seven cycles of combination chemotherapy with capecitabine, cisplatin, and T-mab. Subsequently, he underwent open total gastrectomy, distal pancreatosplenectomy, and extended left hepatic lobectomy as a conversion surgery. The surgically resected specimen demonstrated both adenocarcinoma and neuroendocrine components; therefore, it was diagnosed as HER2-negative mixed adenoneuroendocrine carcinoma. Although the patient received additional chemotherapy, multiple liver metastases appeared at 3 months postoperatively and he died at 6 months postoperatively because of the rapidly progressing metastatic tumor.

**Conclusions:**

We encountered a rare case of rapidly progressive mixed adenoneuroendocrine carcinoma that was negative for HER2 expression after T-mab treatment combined with chemotherapy.

## Background

Although multiple new chemotherapeutic agents have become available, advanced gastric cancer continues to be associated with a high mortality rate; the 5-year survival rates of patients with advanced gastric cancer remain at 20–30% worldwide [1]. Since the publication of the ToGA trial, trastuzumab (T-mab)-based chemotherapy has been considered a standard treatment for HER2-positive advanced gastric cancer [2].

In a systematic review of studies regarding breast cancer, van de Ven et al. demonstrated that the statuses of HER2 and other hormonal receptors changed in 43–51% of patients who received T-mab-containing therapy [3]. Parinyanitikul et al. reported that changes in receptor status after neoadjuvant chemotherapy were associated with improved survival in patients with triple-negative breast cancer [4]. In studies of gastric cancer, several reports demonstrated that HER2 positivity in resected specimens was lost after T-mab-containing chemotherapy in patients with HER2-positive gastric cancer [5, 6]. However, the pathological features which cause loss of HER2 positivity after T-mab therapy remain unknown in gastric cancer.

Herein, we report a rare case of mixed adenoneuroendocrine carcinoma (MANEC) with loss of HER2 positivity after T-mab-based chemotherapy for HER2-positive gastric cancer.

## Case presentation

**Fig. 1:**
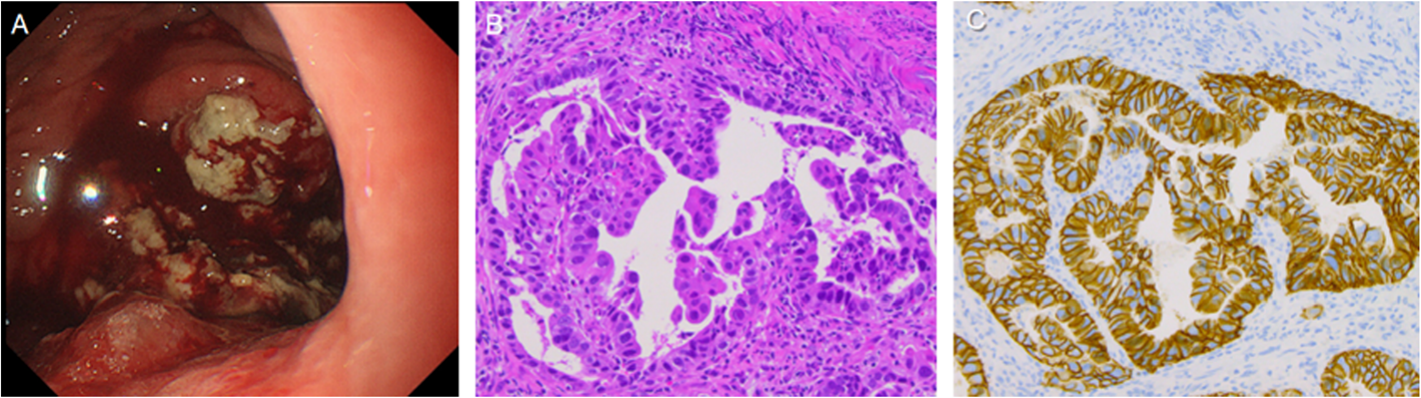
Gastroscopy findings and pathological examinations of biopsy specimen. **a** An ulcerative mass measuring 40–50 mm on the greater curvature corpus of the stomach before chemotherapy. **b** Pathological examination of a biopsy specimen revealed moderately differentiated tubular adenocarcinoma. **c** Immunohistochemistry staining showed strong HER2 expression (3+)

**Fig. 2:**
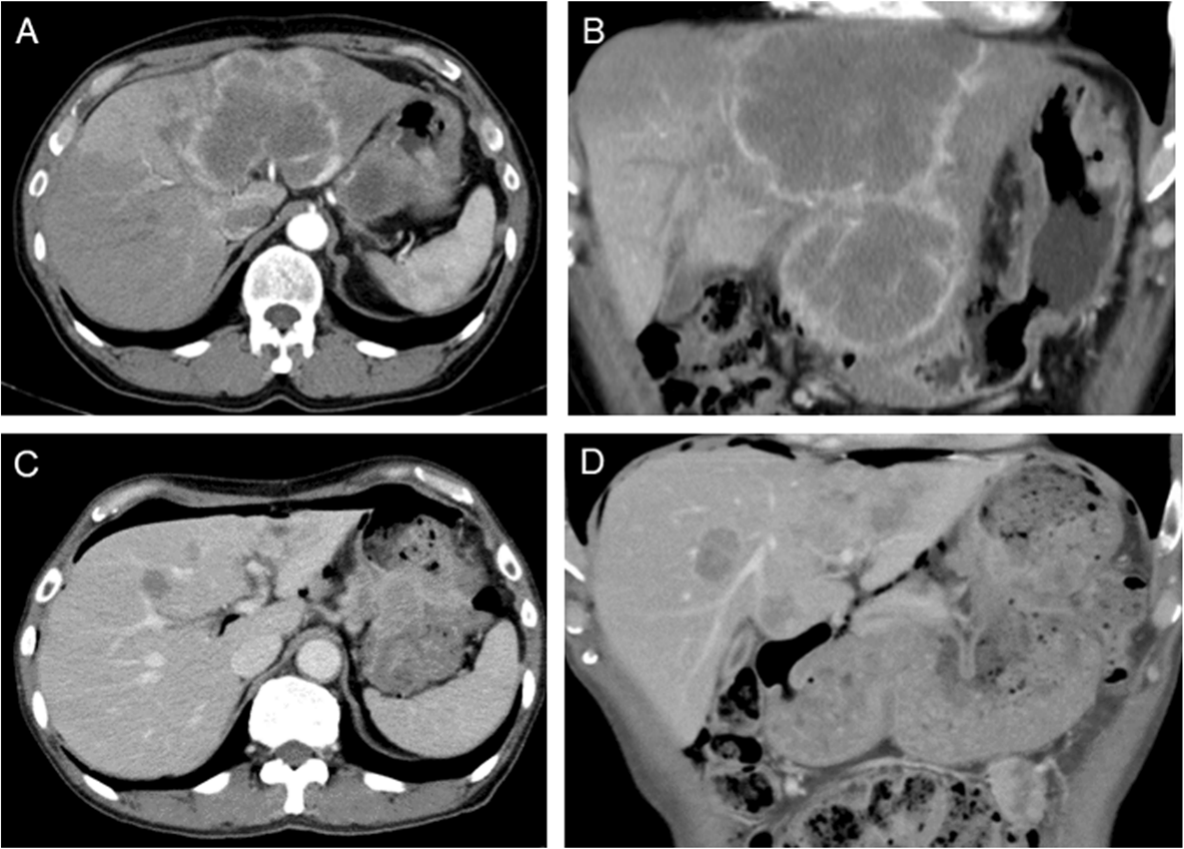
Computed tomography findings. Imaging before chemotherapy revealed thickening in the wall of the stomach and ring-enhanced liver metastases (100 mm and 105 mm in diameter). **a** Axial. **b** Coronal. Imaging after chemotherapy revealed that the stomach tumor and liver metastases had shrunken considerably. **c** Axial. **d** Coronal

A 60-year-old man presented with a mass in the upper abdomen. Blood analysis revealed slight elevations of aspartate aminotransferase (AST, 144 IU/L) and alanine aminotransferase (ALT, 101 IU/L) levels. Serum tumor marker studies showed elevated levels of carcinoembryonic antigen (CEA, 49.3 ng/mL; reference range, < 5.3 ng/mL), carbohydrate antigen 19-9 (CA19-9, 45.0 U/mL; reference range, < 35 U/mL), and alpha-fetoprotein (AFP, 173.2 ng/mL; reference range, < 10 n/mL). Gastroscopy revealed an ulcerative mass measuring 40–50 mm in size on the greater curvature corpus of the stomach (Fig. 1a). Histopathological examination of a biopsy specimen showed moderately differentiated tubular adenocarcinoma and strong (3+) HER2 positivity on immunohistochemical staining (Fig. 1b, c). Enhanced computed tomography revealed wall thickening from the upper to middle stomach and increasing ambient fat concentration. In addition, there were ring-enhanced liver metastases (100 mm and 105 mm in diameter) in the left lobe of the liver; marked enlargement was observed in the left gastric artery and celiac artery lymph nodes (Fig. 2a, b). ^18^F-fluorodeoxyglucose positron emission tomography/computed tomography showed abnormal accumulations of fluorodeoxyglucose at celiac artery lymph nodes, tumors of the upper stomach, and in the left lobe of the liver. Thus, the patient was diagnosed with advanced gastric cancer, T4a(SE) N2 M1(HEP), stage IV.

Because of the HER2-positive immunohistochemical staining results, combination chemotherapy was initiated: capecitabine (2000 mg/m^2^ orally, days 1–14), cisplatin (80 mg/m^2^ intravenously, day 1), and T-mab (6 mg/kg intravenously, day 1). After six courses of chemotherapy, tumor markers, AST, and ALT levels were decreased (CEA, 10.9 ng/mL; CA19-9, 16.3 U/mL; AFP, 72.6 ng/mL; AST 43 IU/L; ALT, 16 IU/L). Computed tomography imaging revealed an extremely shrunken hepatic lesion and lymph nodes (Fig. 2c, d). After seven courses of chemotherapy, the patient experienced severe appetite loss and refused further chemotherapy.

Because laparoscopic exploration did not reveal any unresectable factors, such as peritoneal dissemination or positive cytology, the patient underwent open total gastrectomy (D2 lymph node dissection), distal pancreatosplenectomy, and extended left hepatic lobectomy as a conversion surgery with curative intent. The patient was discharged at 12 days postoperatively with an uneventful postoperative course.

**Fig. 3:**
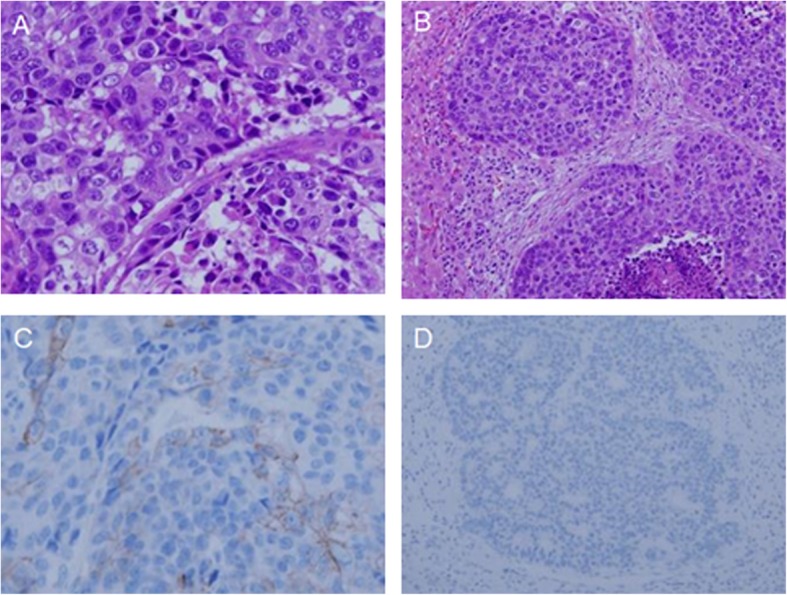
Microscopic examination of the stomach and liver of the resected specimen. Histopathological examination of the primary gastric cancer revealed that it was composed of enriched solid and small nested growths of high nuclear cytoplastic ratio tumor cells with necrosis and fibrosis, which had atypical enlarged hyperchromatic nuclei (**a**). Histopathological examination of the liver showed that the tumors were pathologically compatible with metastases from the gastric cancer (**b**). The immunohistochemical HER2 scores were 1 for the primary gastric cancer and 0 for the liver metastases (primary gastric cancer (**c**), liver metastasis (**d**))

**Fig. 4:**
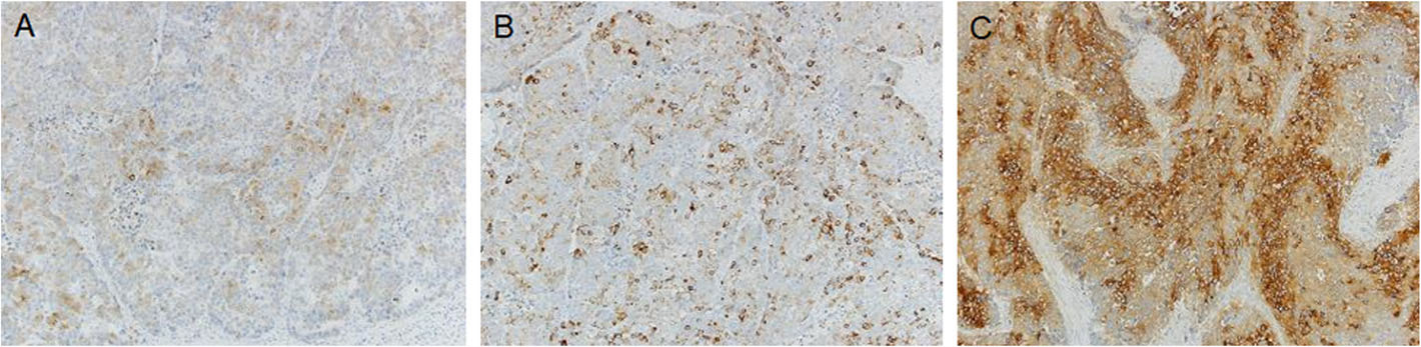
Immunohistochemical staining for synaptophysin, chromogranin A, and carcinoembryonic antigen in the resected specimen. The specimen was positive for synaptophysin (**a**), chromogranin A (**b**), and carcinoembryonic antigen (**c**)

The resected specimen revealed a type 3 tumor on the upper corpus and the greater curvature of the stomach, which measured 85 × 40 mm. The pathological examination revealed that the tumor involved subserosa layer and 2 of 47 lymph nodes were positive for metastasis. Histopathological examination showed that it was composed of enriched solid and small nested growths of high nuclear cytoplastic ratio tumor cells with necrosis and fibrosis; these cells had atypical enlarged hyperchromatic nuclei. In addition, the tumor was composed of adenocarcinoma cells that exhibited tubular growth with cribriform structure (Fig. 3a, b). Immunohistological analysis demonstrated that the tumor cells were focally positive for synaptophysin, chromogranin A, and carcinoembryonic antigen (Fig. 4). The final pathological diagnosis was MANEC. The liver tumors were white solid nodules that measured 66 × 41 × 71 mm in segment 3, 23 × 22 × 17 mm in segments 4 and 8, and 17 × 14 × 15 mm and 23 × 20 × 20 mm in segment 4 of the liver. The tumors were pathologically compatible with metastases from the gastric cancer. The therapeutic effects of chemotherapy were grade 1a. The immunohistochemical HER2 scores were 1 for the primary gastric cancer and 0 for the liver metastases (Fig. 3c, d).

**Fig. 5:**
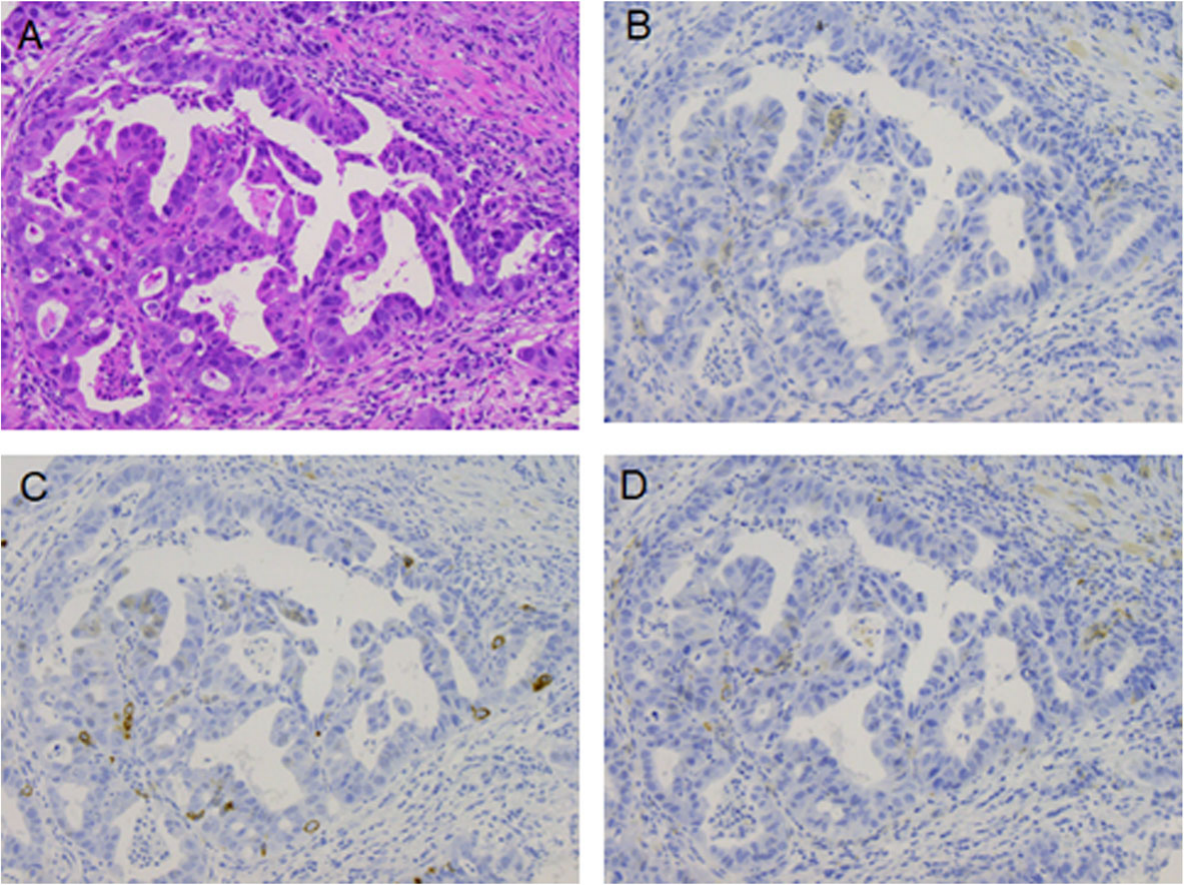
Re-evaluation of the biopsy specimen before surgery of hematoxylin eosin (**a**), synaptophysin (**b**), chromogranin A (**c**), and CD56 (**d**)

After the operation, the patient began to take tegafur-gimeracil-oteracil potassium (S-1; 100 mg/day orally) as adjuvant chemotherapy; we then reduced the dose of S-1 to 80 mg/day beginning in the third course because of the development of grade 1 diarrhea, grade 2 neutropenia, grade 3 thrombocytopenia, and grade 3 terminal ileal inflammation. At 3 months postoperatively, computed tomography imaging revealed four scattered liver metastases, the largest of which was 47 mm in diameter. The patient discontinued adjuvant chemotherapy with S-1 and began chemotherapy with paclitaxel (80 mg/m^2^). After two courses of chemotherapy, computed tomography imaging revealed multiple progressive liver metastases. One month after the end of chemotherapy, the patient exhibited consciousness disturbance due to hepatic disfunction; he died 6 months postoperatively. The autopsy was not performed because the consent was not obtained from the family. We re-evaluated the immunohistochemical status of endoscopic biopsy specimens before surgery; these were negative for synaptophysin and partially positive for chromogranin A and CD56 (Fig. 5).

## Discussion

Based on the results of the ToGA trial published in 2010 [7], T-mab-based chemotherapy was regarded as a standard treatment for HER2-positive unresectable and recurrent gastric cancer. In addition, a randomized phase II trial of T-mab as neoadjuvant chemotherapy for HER2-positive advanced gastric cancer is ongoing [8]. T-mab-based chemotherapy will be frequently performed prior to surgery depending on the results of this trial.

Synchronous liver metastasis of gastric cancer is diagnosed as stage IV disease, and most affected patients are indicated for chemotherapy; however, several patients exhibited good long-term prognosis following radical surgery with hepatectomy [9]. According to the Japanese gastric cancer treatment guidelines published in 2018, if there are only a few liver metastases and no other systemic metastases, surgical resection is weakly recommended for the primary tumor and liver lesions [10]. For the patient in the present case, T-mab combined chemotherapy was extremely effective and no other metastatic site was detected; therefore, conversion surgery was performed with curative intent.

In the World Health Organization classification published in 2010 [11], MANEC was defined as a tumor containing both exocrine and endocrine components, with each component exceeding 30% of the total tumor area. Neuroendocrine carcinoma (NEC) is known to frequently involve lymph node, lymphovascular lumen, and hepatic metastases [12]. Sorbye et al. reported that 2-year and 3-year survival rates of NEC were 14% and 9.5%, respectively, for chemotherapy-treated patients [13]. Thus, gastric NEC exhibits a worse outcome than that of conventional adenocarcinoma [14, 15]. When the biopsy specimen after chemotherapy demonstrates the changes of HER2 expression or histology, it should be meaningful to perform immunohistochemical examinations for NEC to avoid unnecessary surgery. The more aggressive components of NEC should be the target of treatment. Okita et al. proposed the use of irinotecan plus cisplatin as an effective regimen for gastric NEC [16]. However, because of the rarity of this tumor, an optimal treatment regimen remains undetermined.

We re-evaluated the immunohistochemical status of endoscopic biopsy specimens before surgery; these were negative for synaptophysin and partially positive for chromogranin A and CD56, suggesting the difficulty of detecting NEC components in a preoperative endoscopic biopsy specimen. In the present case, the tumor was moderately differentiated adenocarcinoma with HER2-positive staining by preoperative endoscopic biopsy; however, it was finally diagnosed as MANEC due to the loss of HER2 positivity in surgically resected specimens. Thus, we speculated that HER2-based chemotherapy may eliminate HER2-positive adenocarcinoma components; aggressive NEC components without HER2 positivity, which are resistant to HER2-based chemotherapy, became conspicuous [17]. In breast cancer, Guarneri et al. reported that the loss of HER2 expression was more frequently observed in patients with neoadjuvant chemotherapy without T-mab as compared to that in patients with T-mab containing neoadjuvant chemotherapy [18]. We speculated the reason of these conflicting results as follows. First, there was an organ-specific response of HER2 status after T-mab-containing chemotherapy. Second, there were significant differences in the expression pattern of HER2 between gastric and breast cancers, such as the HER2 positivity and its heterogeneity. Lastly, anthracycline, which has been frequently used for breast cancer but not for gastric cancer, was reported to reduce HER2 gene amplification.

In conclusion, we have described our encounter with a rare case of rapidly progressive MANEC that exhibited loss of HER2 expression after T-mab treatment combined with chemotherapy. This consideration is important because T-mab-based chemotherapy is increasingly performed prior to surgery in gastric cancer, and an optimal treat regimen remains undetermined for patients with NEC, such as MANEC.

## Data Availability

All data regarding this paper are available on request.

## Abbreviations

CT: Computed tomography
HER2: Human epithelial growth factor 2
MANEC: Mixed adenoneuroendocrine carcinoma
NEC: Neuroendocrine carcinoma
T-mab: Trastuzumab

## References

[1] Yazici O, Sendur MA, Ozdemir N, Aksoy S (2016). Targeted therapies in gastric cancer and future perspectives. World J Gastroenterol.

[2] Bang YJ, Van Cutsem E, Feyereislova A (2010). Trastuzumab in combination with chemotherapy versus chemotherapy alone for treatment of HER2-positive advanced gastric or gastro-oesophageal junction cancer (ToGA): a phase 3, open-label, randomised controlled trial. Lancet.

[3] van de Ven S, Smit VT, Dekker TJ (2011). Discordances in ER, PR and HER2 receptors after neoadjuvant chemotherapy in breast cancer. Cancer Treat Rev.

[4] Parinyanitikul N, Lei X, Chavez-MacGregor M (2015). Receptor status change from primary to residual breast cancer after neoadjuvant chemotherapy and analysis of survival outcomes. Clin Breast Cancer.

[5] Ikari N, Nakajima G, Taniguchi K (2014). HER2-positive gastric cancer with paraaortic nodal metastasis successfully resected after chemotherapy with trastuzumab: a case report. Anticancer Res.

[6] Ishimine Y, Goto A, Watanabe Y (2015). Loss of HER2 positivity after trastuzumab in HER2-positive gastric cancer: is change in HER2 status significantly frequent?. Case Rep Gastrointest Med.

[7] Sawaki A, Ohashi Y, Omuro Y (2012). Efficacy of trastuzumab in Japanese patients with HER2-positive advanced gastric or gastroesophageal junction cancer: a subgroup analysis of the Trastuzumab for Gastric Cancer (ToGA) study. Gastric Cancer.

[8] Kataoka K, Tokunaga M, Mizusawa J (2015). A randomized Phase II trial of systemic chemotherapy with and without trastuzumab followed by surgery in HER2-positive advanced gastric or esophagogastric junction adenocarcinoma with extensive lymph node metastasis: Japan Clinical Oncology Group study JCOG1301 (Trigger Study). Jpn J Clin Oncol.

[9] Tsujimoto H, Ichikura T, Ono S (2010). Outcomes for patients following hepatic resection of metastatic tumors from gastric cancer. Hepatol Int.

[10] Japanese Gastric Cancer Association. Japanese gastric cancer treatment guideline 2014 (ver. 4). Gastric Cancer. 2017;20:1-19. 10.1007/s10120-016-0622-4 PMC5215069 27342689

[11] Bosman FT, Carneiro F, Hruban RH et al. WHO classification of tumours of the digestive system, vol. 3. 4th ed. Lyon: Interna-tional Agency for Research on Cancer, 2010.

[12] Park JY, Ryu MH, Park YS (2014). Prognostic significance of neuroendocrine components in gastric carcinomas. Eur J Cancer.

[13] Sorbye H, Welin S, Langer SW (2013). Predictive and prognostic factors for treatment and survival in 305 patients with advanced gastrointestinal neuroendocrine carcinoma (WHO G3): the NORDIC NEC study. Ann Oncol.

[14] Xie JW, Lu J, Wang JB (2018). Prognostic factors for survival after curative resection of gastric mixed adenoneuroendocrine carcinoma: a series of 80 patients. BMC Cancer.

[15] Tanaka T, Kaneko M, Nozawa H (2017). Diagnosis, assessment, and therapeutic strategy for colorectal mixed adenoneuroendocrine carcinoma. Neuroendocrinology.

[16] Okita NT, Kato K, Takahari D (2011). Neuroendocrine tumors of the stomach: chemotherapy with cisplatin plus irinotecan is effective for gastric poorly-differentiated neuroendocrine carcinoma. Gastric Cancer.

[17] Mittendorf EA, Wu Y, Scaltriti M (2009). Loss of HER2 amplification following trastuzumab-based neoadjuvant systemic therapy and survival outcomes. Clin Cancer Res.

[18] Guarneri V, Dieci MV, Barbieri E (2013). Loss of HER2 positivity and prognosis after neoadjuvant therapy in HER2-positive breast cancer patients. Ann Oncol.

